# Late Hepatocellular Carcinoma Occurrence in Patients Achieving Sustained Virological Response After Direct-Acting Antiviral Therapy: A Matter of Follow-Up or Something Else?

**DOI:** 10.3390/jcm13185474

**Published:** 2024-09-14

**Authors:** Alessandro Perrella, Alfredo Caturano, Ilario de Sio, Pasquale Bellopede, Adelaide Maddaloni, Luigi Maria Vitale, Barbara Rinaldi, Andrea Mormone, Antonio Izzi, Costanza Sbreglia, Francesca Futura Bernardi, Ugo Trama, Massimiliano Berretta, Raffaele Galiero, Erica Vetrano, Ferdinando Carlo Sasso, Gianluigi Franci, Raffaele Marfella, Luca Rinaldi

**Affiliations:** 1 VII Department of Infectious Disease and Immunology, Ospedali dei Colli, P.O. D. Cotugno, 80131 Naples, Italypasquale.bellopede@ospedalideicolli.it (P.B.);; 2 Department of Advanced Medical and Surgical Sciences, University of Campania “Luigi Vanvitelli”, 80138 Naples, Italyraffaele.galiero@unicampania.it (R.G.);; 3 Gastroenterology Unit, Department of Precision Medicine, University of Campania “Luigi Vanvitelli”, 80138 Naples, Italy; 4 Department of Experimental Medicine, Section of Pharmacology, University of Campania “Luigi Vanvitelli”, 80138 Naples, Italy; 5 Department of Emergency Infectious Diseases and Infectious Diseases, Ospedali dei Colli, P.O. D. Cotugno, 80131 Naples, Italy; 6 Regional Direction for Health Management, Pharmaceutical Unit, 80131 Naples, Italy; 7 Department of Clinical and Experimental Medicine, University of Messina, 98122 Messina, Italy; massimiliano.berretta@unime.it; 8 Department of Medicine, Surgery and Dentistry “Scuola Medica Salernitana”, University of Salerno, 84081 Baronissi, Italy; gfranci@unisa.it; 9 Department of Medicine and Health Science, “V. Tiberio”, Università Degli Studi del Molise, 86100 Campobasso, Italy

**Keywords:** hepatocellular carcinoma, direct acting antivirals, HCV, sustained virological response, liver stiffness, late occurrence, liver disease

## Abstract

**Background**: Despite achieving a sustained virological response (SVR) with direct-acting antivirals (DAAs), an unexpected increase in the occurrence rate of hepatocellular carcinoma (HCC) has been observed among HCV-treated patients. This study aims to assess the long-term follow-up of HCV patients treated with DAAs who achieved an SVR to investigate the potential for late-onset HCC. **Methods**: In this prospective multicenter study, we enrolled consecutive HCV patients treated with DAAs following Italian ministerial guidelines between 2015 and 2018. Exclusion criteria included active HCC on imaging, prior HCC treatment, HBV or HIV co-infection, or liver transplant recipients. Monthly follow-ups occurred during treatment, with subsequent assessments every 3 months for at least 48 months. Abdominal ultrasound (US) was performed within two weeks before starting antiviral therapy, supplemented by contrast-enhanced ultrasonography (CEUS), dynamic computed tomography (CT), or magnetic resonance imaging (MRI) to evaluate incidental liver lesions. **Results**: Of the 306 patients completing the 48-months follow-up post-treatment (median age 67 years, 55% male), all achieved an SVR. A sofosbuvir-based regimen was administered to 72.5% of patients, while 20% received ribavirin. During follow-up, late-onset HCC developed in 20 patients (cumulative incidence rate of 6.55%). The pattern of HCC occurrence varied (median diameter 24 mm). Multivariate and univariate analyses identified liver stiffness, diabetes, body mass index, and platelet levels before antiviral therapy as associated factors for late HCC occurrence. **Conclusions**: Our findings suggest that late HCC occurrence may persist despite achieving SVR. Therefore, comprehensive long-term follow-up, including clinical, laboratory, and expert ultrasonography evaluations, is crucial for all HCV patients treated with DAAs.

## 1. Introduction

Hepatitis C, along with its associated consequences, poses a substantial burden on global public health, healthcare systems, and economies. The World Health Organization (WHO) reported that in 2016, 399,000 individuals succumbed to chronic viral hepatitis, predominantly due to complications, such as liver cirrhosis and liver cancer [1].

In recent years, the treatment landscape for Hepatitis C virus (HCV) infection has undergone a significant transformation with the introduction of new direct-acting antivirals (DAAs). The first generation of DAAs, including protease inhibitors like telaprevir and boceprevir, marked a significant improvement but still required combination with interferon and ribavirin. Recently, more advanced DAAs have been developed, targeting various stages of the HCV life cycle with enhanced efficacy and tolerability. Key novel DAAs include NS5A inhibitors, such as ledipasvir and velpatasvir, NS5B polymerase inhibitors like sofosbuvir, and NS3/4A protease inhibitors, such as grazoprevir and glecaprevir [2]. Combination therapies, like ledipasvir/sofosbuvir, Epclusa sofosbuvir/velpatasvir, and glecaprevir/pibrentasvir, offer highly effective sustained viral response (SVR) rates exceeding 90%, minimal adverse events and a short treatment duration, as demonstrated in clinical trials [3,4,5]. However, concerns have emerged regarding hepatocellular carcinoma (HCC) occurrence in cirrhotic patients achieving an SVR, with conflicting reports indicating both an elevated [6,7,8] and lower risk [9,10].

Among individuals with HCV infection and cirrhosis, the annual risk of HCC is estimated to range from 3% to 7% [11,12]. Earlier studies indicated that cirrhotic patients achieving SVR through interferon (IFN) treatment had a reduced risk of HCC development, with an annual incidence rate of 1.2–1.4% [13,14]. Nevertheless, the risk of HCC persists because antiviral treatment may not completely resolve advanced fibrosis or cirrhosis, which are significant risk factors for liver cancer [15]. Thus, even if these new antivirals have demonstrated their great value in terms of treatment for Hepatitis C eradication in the future, several unresolved issues warrant an evaluation, particularly the following:

(1) Does viral clearance truly signify disease resolution? Clarifying this aspect is crucial for assessing the substantial long-term impact on the natural history of HCV.
(2) Despite more than 5 years of follow-up, uncertainties persist regarding the long-term clinical evolution of viral disease beyond 10 years. Real-life settings lack decisive findings on outcomes, such as ascites, variceal bleeding, hepato-renal syndrome, hepatic encephalopathy, and hepatocellular carcinoma. Addressing these gaps is fundamental for effective healthcare system management.

Building on our prior research protocol on the long-term follow-up of patients undergoing first-generation DAAs [16], this study undertakes a 48-month real-life follow-up to investigate the natural history of the disease in individuals treated with second-generation DAAs, who have achieved an SVR.

## 2. Methods

### 2.1. Study Design and Patient Population

From February 2015 to December 2018, a collaborative effort involving four Hospital and Academic Centres in Southern Italy (Campania Region) on behalf of CLEO (Italian Society of Hospital Hepatologists) conducted an observational, prospective, real-life study assessing the efficacy and safety of DAA treatment regimens. The study enrolled all consecutive HCV patients treated with IFN-free DAA regimens. According to Italian ministerial guidelines for DAA treatment, the inclusion criteria included HCV-RNA serum positivity and fibrosis stage ≥F3 according to the Metavir score. The Italian reimbursement criteria, applicable at the time of enrollment, were limited to patients with F3-F4 fibrosis, assessed via liver biopsy or transient elastography (TE) using Fibroscan^®^ (Echosens, Paris, France). Only TE tests meeting Boursier’s criteria for reliability were considered optimal for enrollment [17].

All individuals meeting the inclusion criterion of undergoing monthly follow-ups during treatment and thereafter every 3 months for a minimum of 48 months were eligible for this real-life population study. Exclusion criteria encompassed individuals with active HCC on imaging or a history of previously treated HCC, those with HBV or HIV co-infection, autoimmune hepatitis, drug-related liver cirrhosis, and liver transplant recipients.

The baseline HCC screening for all patients enrolled in our cohort was performed according to the European guidelines of the European Association for the Study of Liver (EASL) [18].

Before initiating antiviral therapy, abdominal ultrasound (US) was performed within two weeks by two experienced operators at each center, each certified with more than 5000 exams by the Italian Society of Ultrasound Medicine (SIUMB). This approach was used to mitigate potential biases inherent to a multicenter, unmonitored study. Contrast-enhanced ultrasonography (CEUS), dynamic computed tomography (CT) scans, or dynamic magnetic resonance imaging (MRI) was employed to characterize incidental hepatic lesions. Patients exhibiting nodular patterns suggestive of HCC or uncertain dynamic vascular behaviour at treatment initiation were excluded from further follow-up.

HCV-RNA was assessed using real-time PCR (COBAS^®^TaqMan, AmpliPrep, Roche, Basel, Switzerland) with a detection limit of 15 IU/mL.

The diagnosis of cirrhosis relied on clinical, biochemical, ultrasonographic, elastographic, and histological features when available, with liver function graded according to the Child–Turcotte–Pugh (CTP) score system for cirrhotic patients.

Baseline demographic characteristics and clinical parameters, including age, sex, body mass index (BMI), alcohol consumption, tobacco smoking, presence of comorbidities, and biochemical parameters were recorded.

The study was conducted in accordance with the Declaration of Helsinki and later amendments and was approved by the local Ethics Committee, University of Campania Luigi Vanvitelli, 22 December 2017 protocol number 674. All patients provided written informed consent to participate in the study.

### 2.2. Antiviral Treatment

The eligibility of patients for HCV treatment with IFN-free DAA regimens was determined based on priority criteria established in February 2015 by the national Scientific Society and registry of the Italian Medicines Agency Committee (AIFA) [16]. Prescribing clinicians, following national and international guidelines and updates [19,20], individually selected the treatment regimen. The duration of treatment (12 or 24 weeks) was determined based on the severity of liver disease, with longer durations reserved for cirrhotic patients.

Treatment regimens included sofosbuvir+ribavirin, simeprevir+sofosbuvir ± ribavirin, daclatasvir + sofosbuvir ± ribavirin, or ledipasvir + sofosbuvir ± ribavirin or ombitasvir/paritaprevir/ritonavir + dasabuvir ± ribavirin (3D). Therapeutic regimens were categorized as sofosbuvir (SOF)-based, ribavirin (RIB)-included, and sofosbuvir+ribavirin. The ribavirin dosage was never reduced below 600 mg per day in any therapeutic schedule based on adverse events.

### 2.3. Patients Follow-Up

Virological response to therapy was assessed using real-time PCR with HCV-RNA detection at the end of treatment, 12 and 24 weeks after the end of treatment. SVR, defined as the persistent absence of detectable serum HCV-RNA 12 weeks after the end of treatment (SVR12), was evaluated for all the enrolled patients, and any relapse of serum HCV-RNA during follow-up was recorded.

At least three ultrasound examinations per year were performed for every enrolled patient during the established follow-up period (at the end of therapy, 12 weeks after the end of therapy, and 6 months later) in accordance with the HCC surveillance program and study design. Any detected liver lesion underwent an evaluation through imaging technique workup (CEUS, or dynamic CT scan or dynamic MRI) following EASL guidelines [18].

Diagnosed HCC cases were recorded and staged according to the Barcelona Clinic Liver Cancer (BCLC) staging system [19], leading to the cessation of follow-up for patients with an HCC diagnosis.

### 2.4. Liver Stiffness Evaluation

TE by FibroScan, utilizing the M probe (3.5 MHz) with experienced operators (>1000 exams), was conducted following the manufacturer’s instructions. The probe was placed on the skin in the intercostal space over the right lobe of the liver with the patient fasting for at least 3 h. Liver stiffness was calculated over at least 10 valid measurements. The LSM, expressed in Kilopascal (Kpa) within the range of 2.5–75, was assessed for reliability using the interquartile range (IQR)/median ratio (IQR/M) (≤30%). The IQR served as an index of the intrinsic variability of LSM. Moreover, operators were blinded to the clinical and biochemical data of the patients. The IQR corresponds to the interval of LSM results containing 50% of the valid measurement between the 25th and 75th percentiles. Advanced fibrosis was defined as a FibroScan ≥10 kPa but <14 kPa. Cirrhosis was defined as a FibroScan ≥14 kPa in combination with clinical, laboratory, and ultrasound parameters.

### 2.5. Endpoints of the Study

The primary endpoint was to evaluate the late occurrence rate of HCC in HCV patients with SVR after the DAA treatment schedule, defined as carcinoma onset after 48 months from SVR. The secondary endpoint was to evaluate the risk factors associated with late HCC occurrence. Furthermore, a sub-analysis specifically targeted cirrhotic patients, with the primary objective of mitigating potential selection bias and evaluating the distinctions between individuals classified as Child A and Child B according to the Child–Turcotte–Pugh scoring system.

## 3. Statistical Analysis

Data for continuous variables are presented as either the median or range, while categorical variables are expressed as numbers and percentages. Fisher’s exact test or the Chi-square test was employed for analyzing differences between groups in the case of categorical variables. The Mann–Whitney U test or Kruskal–Wallis test was utilized for comparing continuous variables.

For multivariate analysis, logistic regression with stepwise Wald statistic input was performed. Additionally, a Receiver Operating Characteristic (ROC) curve analysis was constructed to assess the actual risk of HCC development based on liver stiffness values (kPa).

Statistical significance was considered for p values below 0.05. All statistical analyses were carried out using the SPSS software (IBM, Armonk, NY, USA), version 24.

## 4. Results

Of the 452 patients initially treated, 306 were successfully followed up for at least 48 months post-treatment, as per the study design. Among the 146 excluded patients, 80 had incomplete data and/or follow-up post-treatment, 30 had HBV coinfection, 23 patients had HIV coinfection, 12 patients had active HCC on imaging or a history of previously treated HCC, 1 died during treatment with causes unrelated to DAAs, and 0 patients suffered from autoimmune hepatitis and/or drug related cirrhosis.

Data distribution was uniform across the participating Centers. The baseline demographic characteristics of the enrolled population are reported in Table 1.

**Figure 1:**
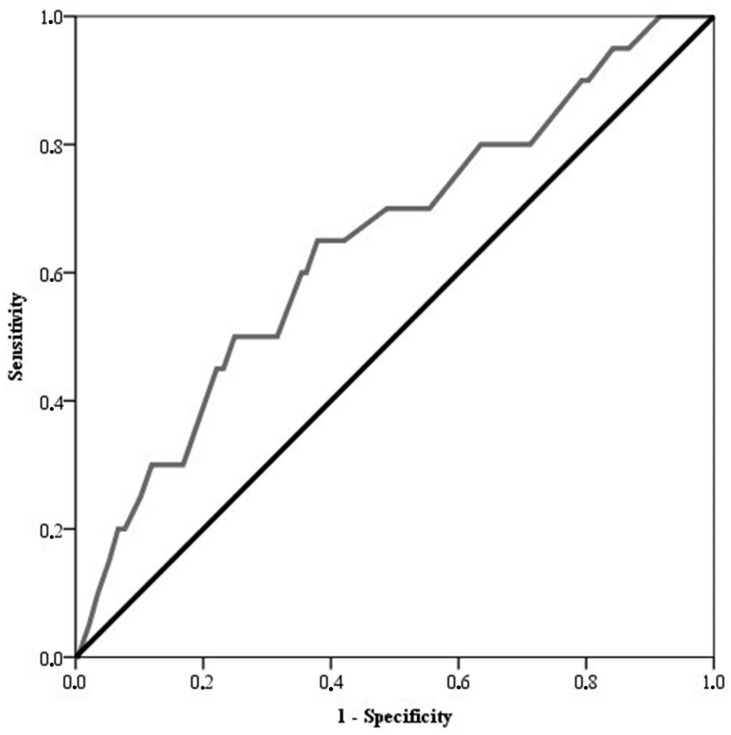
ROC curve describing the discriminant power of the liver stiffness value (kPa) based on the risk of developing late HCC in cirrhotic patients [*n* = 306, AUROC score = 0.646, 95% C.I.: 0.519–0.774]. The *p* value for the significance of liver stiffness based on the risk of HCC was 0.029 (Kruskal–Wallis test).

The median age of the cohort was 67 years, with a predominant male representation (55.1%). Type 2 diabetes was reported in 13.3% of patients. The majority of enrolled patients had a genotype 1 HCV infection (77.8%). The liver stiffness median value was 21 Kpa (range 16–29). A cut-off of 22.9 KPa was associated with a significant increase in the risk of HCC onset (Figure 1). Cirrhosis was present in all patients, with 94.4% classified as CTP class A and 5.6% as class B.

An SOF-based regimen was administered to 72.5% of the patients, 40% of which received ribavirin (26.1% sofosbuvir/ribavirin, 7.2% sofosbuvir/ledipasvir, 1.6% sofosbuvir/daclatasvir, 4.9% in sofosbuvir/simeprevir). All therapeutic schedules are included in Table 2. All enrolled patients achieved an SVR at all timepoints according to the materials and methods. During the follow-up, late HCC onset was observed in 20 patients, yielding a cumulative incidence rate of 6.55%. The pattern of HCC occurrence was heterogeneous, with 13 patients exhibiting a nodular profile and 3 developing infiltrative HCC, 4 of which had macro-vascular invasion, including portal vein thrombosis. No patient showed extrahepatic metastases. All patients with HCC occurrence maintained an SVR without any viral relapse. The median diameter of the lesions was 24 mm (range 15–37 mm). None of the HCC patients were active alcohol consumers, and only 8.5% were smokers with an average of 10 cigarettes per day. According to BCLC classification, 13 patients were categorized as stage A, 3 as stage B, and 4 as stage C. Among the enrolled patients, 90% received a SOF-based treatment, and 60% of them were treated without ribavirin. Univariate analysis identified CTP B stage (*p* = 0.001), comorbidity of diabetes (*p* = 0.007), presence of cirrhosis (*p* = 0.002), and the liver stiffness value (*p* = 0.0001) as significantly associated with HCC occurrence. Through multivariate analysis, liver stiffness, diabetes, BMI, and platelet levels before antiviral therapy were identified as factors related to late HCC occurrence (Table 3).

## 5. Discussion

The pathogenesis and natural history of HCV are influenced by several factors, including immune system activity in both regulatory and effector environments from the early phase of infection [20,21]. Chronic infection leads to persistent immune system activity, creating a specific cytokine environment that results in liver necro-inflammation and, over time, may lead to the onset of HCC [22,23], driven by continuous inflammation and related fibrosis [24]. Patients who have achieved a sustained virological response (SVR) following hepatitis C virus (HCV) treatment still face a persistent risk of hepatocellular carcinoma (HCC), particularly those with advanced fibrosis or cirrhosis. Despite SVR being the goal of therapy, studies indicate an annual HCC risk ranging between 1.8% and 2.5% in these populations.

In interferon-based regimens, a significant reduction in HCC incidence, though not its complete elimination in those with viral clearance, has been associated with factors, such as the persistence of a cirrhotic architecture, advanced age, latent mutations of HCV, comorbidities like diabetes, and the consumption of alcohol and tobacco [13,14,25,26]. The recent approval of second-wave DAAs does not yet allow for a long-term assessment of the impact of SVR on HCC incidence. This independent, spontaneous, real-life study on a long-term follow-up aimed to verify the natural history of the disease after viral clearance. Our findings indicate the potential occurrence of late HCC even after successful viral clearance. Intriguingly, this association with neoplastic onset appears to be more prominent among patients classified as CTP class A. This finding suggests that there could be other factors beyond fibrosis that may play a significant role in HCC development in this specific patient subset [27]. Notably, all HCC cases occurred in those treated with an SOF-based antiviral regimen, highlighting that achieving an SVR with DAAs does not completely prevent the occurrence of late HCC. These findings raise questions, as the carcinogenic effect of HCV proteins is traditionally associated with necroinflammation in the presence of active viral replication, which deregulates host cell cycle checkpoints and the virus and immune-mediated oxidative stress leading to DNA mutations in liver cells [28,29,30]. Despite this scientific evidence, mechanisms related to late HCC occurrence are still unclear, even considering possible pathogenetic models that could explain the possible reason for the onset of late HCC in patients with SVR. Indeed, the reason why those who underwent DAA schedules, including SOF, seem to be likely associated with early and late HCC may be related to several factors. These include potential effects on immunosurveillance mechanisms in patients with advanced fibrosis, such as the downregulation of interferon genes during DAA therapy and increased hepatocyte proliferation in the absence of appropriate checkpoints. This mechanism could potentially promote tumor development [21]. While antiviral agents, including SOF, have been instrumental in eradicating HCV and reducing the risk of liver-related complications, like HCC, our findings on late HCC occurrence suggest that there is still a need for further studies. Insights from studies comparing DAA versus interferon-based regimens and untreated cohorts indicate a reduced risk of HCC recurrence with DAA therapy, attributed to the effective suppression of viral replication and subsequent inflammation [22]. Moreover, both viral and host-related factors significantly influence HCC development following HCV RNA eradication [22]. Recent studies have highlighted distinct roles for viral factors, such as HCV genotype-specific mutations, including the HCV-1B core amino acid 70 mutation, which have been associated with an increased risk of HCC post-treatment. Concurrently, host-related factors, such as alpha-fetoprotein levels, have been identified as predictors of HCC occurrence after successful HCV treatment to an SVR [14,24]. These findings underscore the complexity of HCC pathogenesis post-HCV cure, suggesting that variations in HCV genotypes, subtypes, and genetic mutations may contribute to differing HCC risks [24,31]. Moreover, these findings underscore the need for enhanced surveillance of patients who achieve an SVR but exhibit high liver stiffness or metabolic risk factors, as they remain at risk for late-onset HCC [32,33,34]. Therefore, implementing more frequent imaging and metabolic assessments, alongside updating guidelines and educating patients, is crucial for this subset of patients [32,33,34]. Further investigations into the molecular mechanisms underlying these associations are warranted to enhance our understanding and optimize surveillance strategies for patients achieving an SVR.

This prospective multicenter study on the long-term outcomes of HCV patients treated with second-generation DAAs and achieving an SVR possesses several notable strengths. The prospective design enhances the reliability of the data, allowing for a meticulous follow-up and documentation of patients over a 48-month period. The inclusion of consecutive HCV patients treated according to established guidelines reflects real-world clinical practices, enhancing the external validity of the findings. The comprehensive assessment, including clinical, laboratory, and imaging data, provides a robust foundation for analyzing the factors influencing late HCC occurrence post-SVR. While the study holds strengths, certain limitations must be considered. The observational nature of the study introduces potential biases, and the absence of a control group may limit the ability to establish causal relationships. The exclusion of patients with active HCC at baseline may impact the generalizability of the findings to the broader HCV population. The study’s focus on patients achieving SVR may introduce selection bias, and the association between specific DAA regimens and HCC needs further exploration. Moreover, the study’s predominantly Italian population may limit the applicability of its findings to more diverse populations, where genetic and environmental factors influencing hepatocellular carcinoma (HCC) development post-SVR could vary. Ethnic diversity plays a crucial role in shaping genetic predispositions and environmental exposures relevant to HCC. The study’s limited representation of diverse ethnic backgrounds restricts an extrapolation of the findings to populations with differing genetic susceptibilities and environmental risks for HCC [35,36,37]. Despite efforts to account for potential confounders, the study may not comprehensively address all factors influencing HCC development post-SVR. Additionally, the relatively short follow-up duration may not capture the entirety of long-term risks. Despite these limitations, this prospective study provides valuable insights into the real-world implications of SVR on HCC outcomes in HCV patients treated with second-generation DAAs.

## 6. Conclusions

HCC remains a fearsome complication of cirrhosis in HCV, necessitating surgical therapies, including the possibility of liver transplantation, and the use of last-generation drugs [30,31]. Our findings, in accordance with previous evidence, underscore that viral clearance induced by DAA regimens does not eliminate the risk of late HCC onset. Therefore, a comprehensive long-term follow-up, incorporating clinical, laboratory, and expert ultrasonography assessments, should be diligently conducted for these patients. The development of new antivirals should consider potential late adverse events, emphasizing the need for a holistic approach in drug development.

## Figures and Tables

**Table 1:** Baseline characteristics of the entire study cohort (*n* = 306).

Parameter	
Age (yrs.), median [IQR]	67 [60–73]
Sex, *n* (%)	
Male	165 (53.9)
Female	141 (46.1)
BMI, median [IQR]	26.1 [24.3–28]
Smoke, *n* (%)	2 (0.7)
Potus, *n* (%)	2 (0.7)
Diabetes, *n* (%)	59 (19.3)
Metabolic syndrome, *n* (%)	22 (7.2)
Number of lesions, *n* (%)	
0	286 (93.5)
1	15 (4.9)
≥2	5 (1.6)
Portal invasion, *n* (%)	4 (1.3)
Bright liver, *n* (%)	22 (7.2)
Liver stiffness (kPa), median [IQR]	21 [16–29]
Duration of therapy, median [IQR]	12 [12–24]
Platelets, median [IQR]	
T0	108,000 [73,000–153,000]
SVR12	103,500 [65,250–122,000]
Genotype, *n* (%)	
1	243 (79.4)
2	50 (16.3)
3	11 (3.6)
4	2 (0.7)
Child–Pugh Score T0, *n* (%)	
A	289 (94.4)
B	17 (5.6)
Child–Pugh Score SVR12, *n* (%)	
A	298 (97.4)
B	8 (2.6)
Therapy, *n* (%)	
Sofosbuvir/Ribavirin	80 (26.1)
Sofosbuvir/Ledipasvir	69 (22.5)
Sofosbuvir/Daclatasvir	23 (7.5)
Sofosbuvir/Simeprevir	50 (16.3)
3D	84 (27.5)
Late HCC, *n* (%)	20 (6.5)

Data are expressed ad either the number and percentage or median and interquartile range (IQR). BMI, body mass index; SVR, sustained virological response has been observed at all timepoints 12–14–48 weeks; HCC hepatocellular carcinoma

**Table 2:** Therapeutic schedules.

Treatment Regimen	Number of Patients (%)
Sofosbuvir/Ribavirin	80 (26.1%)
Sofosbuvir/Ledipasvir	69 (22.5%)
Sofosbuvir/Daclatasvir	23 (7.5%)
Sofosbuvir/Simeprevir	50 (16.3%)
3D (Ombitasvir/Paritaprevir/Dasabuvir)	84 (27.5%)

**Table 3:** Baseline characteristics of cirrhotic patients according to late HCC development: univariate and multivariate analysis (*n* = 306).

	Univariate Analysis			Multivariate Analysis	
	HCC				
Parameter	Yes (*n* = 20)	No (*n* = 286)	*p*	O.R. [95% C.I.]	*p*
Age (yrs), median [IQR]	70 [68.2–75]	67 [59.5–72]	0.026		
Sex, *n* (%)			0.050		
*M/F*	15 (75)/5 (25)	150 (52.4)/136 (47.6)			
BMI, median [IQR]	25 [23.2–26.7]	26.2 [24.7–28.4]	0.026	0.712 [0.537–0.943]	0.018
Smoke, *n* (%)	2 (10)	0 (-)	0.000		
Potus, *n* (%)	2 (10)	0 (-)	0.000		
Diabetes, *n* (%)	9 (45)	50 (17.5)	0.003	0.180 [0.045–0.713]	0.015
Metabolic syndrome, *n* (%)	3 (15)	19 (6.6)	0.162		
Liver stiffness (kPa), median [IQR]	26.5 [18–44.5]	20.4 [16–28.7]	0.028	1.070 [1.020–1.122]	0.006
Duration of therapy (weeks), median [IQR]	12 [12–12]	12 [12–24]	0.007		
Platelets, median [IQR]					
T0	75,000 [48,000–109,500]	115,000 [80,000–166,000]	0.001	0.975 [0.954–0.996]	0.019
SVR	n.a.	n.a.	n.a.		
Genotype, *n* (%)			0.095		
1	15 (75)	228 (79.7)			
2	3 (15)	47 (16.4)			
3	1 (5)	10 (3.5)		0.007 [0.000–0.417]	0.017
4	1 (5)	1 (0.3)		0.003 [0.000–0.280]	0.012
Number of lesions, *n* (%)			0.000		
0	0 (-)	286 (100)			
1	15 (75)	0 (-)			
≥2	5 (25)	0 (-)			
Portal invasion, *n* (%)	4 (11.4)	0 (-)	0.000		
Bright liver, *n* (%)	1 (5)	21 (16.8)	0.172		
Child–Pugh Score T0, *n* (%)			0.000		
A	15 (75)	274 (95.8)			
B	5 (25)	12 (4.2)			
Child–Pugh Score SVR12, *n* (%)			0.000		
A	17 (85)	281 (98.3)			
B	3 (15)	5 (1.7)			
Therapy, *n* (%)			0.000		
Sofosbuvir/Ribavirin	16 (80)	64 (22.4)			
Sofosbuvir/Ledipasvir	0 (-)	69 (24.1)			
Sofosbuvir/Daclatasvir	1 (5)	22 (7.7)			
Sofosbuvir/Simeprevir	1 (5)	49 (17.1)			
3D	2 (10)	82 (28.7)			

Data are expressed ad either the number and percentage or median and interquartile range (IQR) BMI, body mass index; SVR, sustained virological response has been observed at all timepoints 12–14–48 weeks.

## Data Availability

The data that support the findings of this study are available on reasonable request from the corresponding author.
